# Altered Tumor-Cell Glycosylation Promotes Metastasis

**DOI:** 10.3389/fonc.2014.00028

**Published:** 2014-02-13

**Authors:** Irina Häuselmann, Lubor Borsig

**Affiliations:** ^1^Zürich Center for Integrative Human Physiology, Institute of Physiology, University of Zürich, Zürich, Switzerland

**Keywords:** glycosylation, cancer, metastasis, glycan ligands, mucins, siglecs, galectins, selectins

## Abstract

Malignant transformation of cells is associated with aberrant glycosylation presented on the cell-surface. Commonly observed changes in glycan structures during malignancy encompass aberrant expression and glycosylation of mucins; abnormal branching of *N*-glycans; and increased presence of sialic acid on proteins and glycolipids. Accumulating evidence supports the notion that the presence of certain glycan structures correlates with cancer progression by affecting tumor-cell invasiveness, ability to disseminate through the blood circulation and to metastasize in distant organs. During metastasis tumor-cell-derived glycans enable binding to cells in their microenvironment including endothelium and blood constituents through glycan-binding receptors – lectins. In this review, we will discuss current concepts how tumor-cell-derived glycans contribute to metastasis with the focus on three types of lectins: siglecs, galectins, and selectins. Siglecs are present on virtually all hematopoietic cells and usually negatively regulate immune responses. Galectins are mostly expressed by tumor cells and support tumor-cell survival. Selectins are vascular adhesion receptors that promote tumor-cell dissemination. All lectins facilitate interactions within the tumor microenvironment and thereby promote cancer progression. The identification of mechanisms how tumor glycans contribute to metastasis may help to improve diagnosis, prognosis, and aid to develop clinical strategies to prevent metastasis.

## Introduction

The majority of cancer deaths are attributed to the metastatic spread of cancer cells to vital organs rather than to the primary tumor outgrowth. During malignant transformation, the genetic alteration in the cells results in mutations of proto-oncogenes and tumor suppressor genes, which as a result give rise to tumor clones with different properties (1). Malignant cells thereby acquire characteristics enabling them dissociation from tumors, degradation of the extracellular matrix, invasion, adhesion, and metastasis to distant organs. Alteration of tumor-cell-surface glycosylation is one of the characteristic traits associated with enhanced malignancy (2–4). Glycans are oligosaccharide structures that are covalently bound to proteins, lipids, or present in a free form in tissues or tumors. Glycans are bound to the protein either through Asn (*N*-linked glycan) or through Ser or Thr (*O*-linked glycan). Lectins are a family of carbohydrate-binding proteins that specifically recognize glycans. Fundamental processes such as cell–cell recognition, cell adhesion, mobility, and pathogen–host interaction are facilitated by lectins in healthy organisms. The common expression of lectins on endothelial cells, immune cells, in the extracellular matrix or as soluble adhesion molecules enables them to bind to tumor-cell glycans and thereby affect tumor-cell progression (5). Subsequently, accumulating evidence supports the involvement of tumor-cell-surface glycans in tumor-cell migration, adhesion, and metastasis. This review addresses the role of cancer-associated glycans during metastasis with the focus on endogenous lectin interactions within the tumor microenvironment.

## The Process of Metastasis

Hematogenous metastasis is a multistep process during which malignant cells detach from the primary tumors, degrade the extracellular matrix, invade the surrounding tissue, enter the blood or lymphatic vessels, and extravasate to form metastatic lesions. Tumor cells through the cell-surface glycans can engage with a variety of endogenous lectins both at the primary site of a tumor and in the circulation. Tumor cell upon reaching the blood circulation induces microthrombi, the formation of which is facilitated by platelet P-selectin binding to tumor-cell-surface glycans (6, 7). Tumor-cell emboli formation contributes to mechanical lodging in the microvasculature and/or adhesion to the endothelium thereby promoting tumor-cell extravasation and metastasis (8). There is accumulating evidence that vascular lectins–selectins facilitate tumor-cell interactions with all blood constituents, platelets, leukocytes, and endothelial cells, and thereby contribute to metastasis (3, 9, 10). In addition, recruitment of immune cells to the metastatic microenvironment is dependent on selectins (11–13).

Specific glycan structures on colonic epithelium provide immune-modulatory activity to tissue macrophages through sialic acid-binding lectins–siglecs (14, 15). In addition, galactose-binding lectins–galectins were shown to be involved in immune-suppression and metastasis (16). Consequently, altered glycosylation may both induce inflammatory reactions and promote immune-suppression, however; it is dependent on the cellular context within the tissue. Finally, glycan changes associated with cancer progression profoundly define the phenotype of cancer cells depending on interactions with endogenous lectins both in tumor and metastatic environments.

## General Mechanisms for Altered Glycosylation in Cancer

Cancer progression requires a range of alterations in extracellular and intercellular signaling that promotes cell proliferation, emergence of invasive subsets, dissociation from the tumor, intravasation, and adhesive interactions within the circulation that finally facilitate metastasis. Within the tumor environment changes in glycosylation allow malignant cells to promote cell mobility, cell adhesion, and even receptor activation, and thereby contributing to the invasive phenotype (3–5). Malignant transformation leads to expression of oncofetal antigens, epitopes that are present on embryonic tissues and tumor cells, but are generally absent in healthy adult cells. Neo-synthesis and incomplete synthesis are the two major mechanisms for generation of cancer-specific glycans (2).

Altered glycosylation of *N*-linked glycans in cancer is typically associated with enhanced β1,6-branching (Table 1) that is facilitated by β1,6-*N*-acetylglucosaminyltransferase-5 (GnT5) (17, 18). Increased activity of GnT5 is associated with increased polylactosaminic sequences, and the inhibition of GnT5 resulted in attenuation of metastasis (19, 20). GnT5 deficiency (Mgat5-deficient mice) resulted in reduced tumor growth and metastasis (21). However, the functional role of branched N-glycosylation in cancer was later shown to be dependent on galectin binding and thereby altering the phenotype of the cell (22).

**Table 1:** **Common glycan alterations on carcinoma cells and their effect on lectin recognition**.

Structural change	Carriers	Biosynthetic basis of structural change	Potential lectin partners	Reference
Increased β1,6-branching (*N*-linked)	*N*-glycans	Increased GnT5	Galectins Siglecs	Guo et al. (19), Lagana et al. (20)
Increased α2,6-sialylation	*N*-glycans, e.g., β integrin	Increased ST6Gal1 sialyltransferase		Seales et al. (32)
General increase in sialylation	Mucins *N*-glycans	Increased sialyltransferase activity	Selectins, siglecs, galectins	Dall’Olio et al. (30), Gessner et al. (31)
Increased sialyl-Lewis^x/a^	Mucins	Increased FUT7, FUT3, FUT6, ST3Gal6	Selectins	Barthel et al. (169), Julien et al. (195), Koike et al. (27), Ogawa et al. (161), Yin et al. (198)
Decreased di-sialyl-Lewis^x/a^	Mucins, glycolipids	Decreased ST6GalNAc6	Selectins	Miyazaki et al. (41), Tsuchida et al. (42)
		GlcNAc6ST1	Reduced siglecs binding	Nudelman et al. (43)
Increased Tn epitopes	Mucins (e.g., MUC1), CD44, β1 integrin, osteopontin	Downregulated T-synthase activity due to Cosmc mutations	Galectins	Ju et al. (40)
Increased sialyl-Tn epitopes		Increased ST6GalNAc1 expression	Siglecs	Julien et al. (67), Ozaki et al. (68)
			Galectins	
Increased T antigen (core 1 structure)		Decreased C2GnT2	Galectins	Brockhausen et al. (53), Dalziel et al. (55)
		Enhanced availability of UDP-galactose		Kumamoto et al. (73)
Increased sialyl-T antigens		Increased levels of α2,3-sialyltransferase (ST3Gal1)	Galectins Siglecs	Burchell et al. (78), Dalziel et al. (55), Picco et al. (79), Schneider et al. (72)

Virtually in every cancer type upregulation of glycosyltransferases has been detected, leading to expression of common tumor-cell epitopes such as sialyl-Lewis^x^ and sialyl-Lewis^a^ (sLe^x^/sLe^a^), Thomsen-nouvelle antigen (Tn), and sialyl-Tn (sTn) (3–5, 23, 24). Hypoxia has been identified as one of the factors leading to increased expression of glycosyltransferases (25, 26). For instance, increased expression of α1,3-fucosyltransferase-7 (FUT7) and α2,3-sialyltransferase ST3Gal1, enzymes involved in synthesis of sLe^x/a^ has been detected (27). The general increase in sialylation has been detected both in clinical settings and experimental models that is associated with a metastatic cell phenotype (25, 28, 29). An increase in α2,6-sialylation in tumors is usually attributed to the upregulation of ST6Gal1 sialyltransferase that is primarily active on *N*-linked glycans (30–32), or ST6GalNAc family of sialyltransferases, which are active on *O*-linked glycans or glycolipids (33). Accordingly, overexpression of Neu1 sialidase in colon cancer cells led to reduced liver metastasis in mice due to increased desialylation of β4 integrin whereas silencing of Neu1 sialidase increased cell migration, invasion, and adhesion *in vitro* (34).

Synthesis of shorter glycan structures like Thomsen–Friedenreich (TF or T), Tn, and sTn epitopes has been observed in a number of carcinomas (35–39). One of the factors affecting the synthesis of incomplete glycan structures is the frequent mutation of the *Cosmc* chaperone that is required for the galactosyltransferase activity that modifies *O*-linked glycans (40). Another example of shortened glycan synthesis is the reduced expression of disialyl-Lewis^a^ (di-sLe^a^) and sialyl 6-sulfo Lewis^x^ structures in epithelial cancer. Disialyl-Lewis^x^ (di-sLe^x^) structure is synthesized with the α2,6-sialyltransferase ST6GalNAc6, and its expression is downregulated by epigenetic silencing in malignant epithelium (41, 42). Similarly, repressed expression of sulfotransferase responsible for 6-sulfo Le^x^ was detected in cancer cells but not in normal epithelial cells (26).

Gangliosides are sialic acid-containing glycolipids, which expression is often dysregulated during malignant transformation (2). Apart from glycolipid specific glycan structures containing disialic acid in a α2,8-linkage (e.g., GD3), changes in glycosyltransferases promote expression of sLe^x^ epitopes (43). Overexpression of sialidase Neu2 led to reduced metastasis, while Neu2 was found to be downregulated in highly metastatic variants of colon carcinoma (44).

Despite many possibilities for the formation of glycans (linkage and sequence of monosaccharide units) there is a rather small number of structures commonly detected in cancer. Furthermore, terminal glycan structures exposed on the cell surfaces of tumor cells can be recognized through endogenous lectins and thereby modulate cancer progression.

## Alterations of Cancer-Associated *O*-Linked Glycans

**Figure 1:**
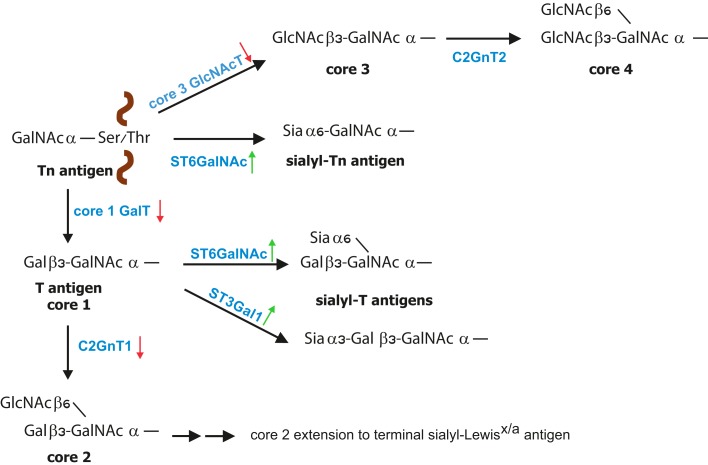
**Biosynthesis of *O*-glycans**. *O*-glycan synthesis is initiated by linking of GalNAc to the protein at Ser or Thr residue. The simplest *O*-glycan Tn antigen can be further converted to core 1 structure (T antigen) by β1,3 galactose extension; core 3 structure by addition of β1,3-GlcNAc. During cancer increased expression (green arrow) of sialyltransferases with concomitant reduced expression (red arrow) of core 1 GalT and core 3 GlcNAcT leads to increased formation of sialyl-Tn and sialyl-T antigens. Core 1 structure is further branched by C2GnT1 to form core 2 that can be further modified to poly-*N*-acetyllactosamine structures carrying sialyl-Lewis^x/a^ [modified from Ref. (23)].

Mucins are high molecular weight glycoproteins exhibiting a rod like conformation due to heavy glycosylation with *O*-linked glycans (3, 45). *O*-linked glycosylation, which is based on GalNAc bound to the Ser/Thr of a protein, is further modified by galactose (core 1 structure) or GlcNAc (core 3 structure) in normal mucins (Figure 1). During malignant transformation mucins of intestine, colon, liver, and pancreas have reduced core 1 and core 3 structures that correlate with enhanced sialylation of Tn and T antigens (24, 46, 47). Core 3-derived glycans are a major type expressed by normal epithelial cells of the gastrointestinal tract, which are downregulated during malignancy due to loss of functional β3-*N*-acetylglucosaminyltransferase-6 (core 3 synthase) expression (48, 49). Consequently, overexpression of core 3 synthase in pancreatic cells was associated with decreased presence of Tn antigens and resulted in a reduced tumorigenicity and metastasis upon orthotopic injection. In addition, enhanced expression of the core 2 β1,6-*N*-acetylglucosaminyltransferase (C2GnT1) responsible for the core 2 synthesis was detected in colorectal and lung carcinomas, which correlated with high levels of sLe^x^ on *O*-glycans and therefore strong binding to E-selectin and metastasis compared to normal tissues (50–52). Mucins of normal mammary epithelial cells contain a mixture of *O*-glycans and the majority is core 2-based structures (53, 54). Reduced expression of C2GnT1 in mammary cancer is associated with enhanced presence of Tn and sTn (53–56). However, despite reduced core 2 structures on breast cancer cells, increased presence of sLe^x^ epitopes has been observed, which likely is a result of increased fucosylation (57).

## Formation of T, Tn, and sTn Antigens during Cancer Progression

In healthy tissues, core 1-based T and Tn epitopes are almost absent however; in about 90% of all human carcinomas these precursor structures are detected (36, 39). Unsubstituted Tn epitopes occur in human cancers of colon, breast, bladder, prostate, liver, ovary, and stomach; and their presence correlate with cancer progression and metastasis (35–37, 58–63). Similarly, sialylated T and Tn antigens correlate with progression of epithelial cancer and poor clinical prognosis of many carcinomas (25, 28, 39, 64–66). ST6GalNAc1-mediated α2,6-linked sialylation of GalNAc of the precursor Tn antigen results in formation of the sTn antigen (25, 67–69). The sialylation step prevents further glycan extension and therefore leads to truncation of *O*-linked glycans (47, 70).

Several mechanisms have been described to enable increased Tn, sTn, or T expression in cancer (Table 1) (33, 46). (1) Decreased activity of core 2 C2GnT1 enzyme leads to accumulation of T antigen (described above) that is further sialylated by ST6GalNAc1 and ST6GalNAc2 enzymes (71, 72). (2) Enhanced availability of the nucleotide sugar substrate UDP-galactose appears to promote increased T antigen biosynthesis through core 1 β1,3-galactosyltransferase (73). Colon cancer tissues expressed increased levels of the UDP-Galactose transporter, which brings the sugar donor into the Golgi apparatus compared to non-malignant mucosa. (3) Activity of β1,3-galactosyltransferase (T synthase) requires the presence of the molecular chaperon protein *Cosmc*, which is responsible for folding and stability of the enzyme (40, 74). The absence of *Cosmc* leads to β1,3-galactosyltransferase degradation. Mutation in *Cosmc* chaperone is associated with increased Tn expression in colon carcinoma and melanoma cell lines and also increased sTn expression (40, 75). Accordingly, down-regulation of T-synthase resulted in a marked increase of T, Tn, and particularly sTn in colon carcinoma cells (76). (4) Generation of sTn is facilitated by the sialyltransferase ST6GalNAc1 and ST6GalNAc2 (71, 72). Human gastric cancer cells with enhanced ST6GalNAc1 expression showed higher intraperitoneal metastasis compared to sTn-negative tumor cells. Similarly, overexpression of ST6GalNAc1, thereby sTn epitope, in human breast cancer cells led to increased tumor growth in immunodeficient mice (68, 77). In addition, enhanced sialylation of T antigen in breast cancer correlated with higher levels of α2,3-sialyltransferase (ST3Gal1) (72, 78). Overexpression of ST3Gal1 under the human MUC1 promoter in a spontaneous murine breast cancer model resulted in significantly decreased tumor latency compared to mice without ST3Gal1 overexpression (79). Furthermore, the sialyltransferase expression alone was responsible for enhanced tumorigenesis indicating that this enzyme *per se* acts as a tumor promoter (79).

Only few glycoproteins are known to present Tn, T, or sTn and sialyl-T (sT) antigens in malignant tissues (66). Mucin MUC1 and CD44v6 display sTn and sT antigens in colon, gastric, and breast cancers (80–83). MUC2 is a major carrier of shortened glycans in gastric cancer (84). Enhanced sTn expression in breast and gastric cancer is associated with overexpression of MUC1, CD44, and ST6GalNAc1 (68, 77). Although CD44v6 is expressed in some types of healthy epithelia, higher expression is observed in squamous cell carcinomas and adenocarcinomas including breast, lung, colon, and pancreatic carcinomas (85–87). Interestingly, serum levels of osteopontin, a CD44 ligand, that itself is a sTn carrier, have been detected in cancer patients and correlate with poor prognosis (87).

The enhanced expression of Tn, sTn, and T antigens on MUC1, osteopontin, and CD44 is associated with high metastatic potential and poor prognosis (84, 88, 89). However, there is little evidence for the functional consequence of this aberrant glycosylation during cancer progression. In human breast cancer cells, expression of sTn on MUC1 was associated with reduced cell adhesion and increased cell migration (77). In addition, β1 integrins carry aberrant forms of *O*-glycans that is associated with metastasis (90). Enhanced expression of ST6GalNAc1 in murine carcinoma cells led to an increase in sTn expression on β1 integrin subunit associated with morphological changes including loss of epithelial appearance, disorganization of actin stress fibers, and reduced ability to migrate on fibronectin. A recent study showed that high expression of the ppGalNAcT13, which initiates *O*-glycan synthesis by adding the first GalNAc to Ser/Thr, induced high metastatic potential of Lewis lung carcinoma by generating trimeric Tn antigens (GalNAc1-Ser/Thr)_3_ on syndecan 1 (91). The complex formation of trimeric Tn antigens on Syndecan 1 together with α5β1 integrin and MMP-9 resulted in enhanced invasion and metastasis. Recent findings provide evidence that cell-surface mucins are involved in signal transduction events [reviewed in Ref. (24, 45)]. Decreased sTn expression on neuroblastoma achieved by extension of core 1 structure with B3GNT3 expression reduced activation of focal adhesion kinase and thereby partially suppressed malignant phenotype (92). Aberrant glycosylation in cancer does not affect only the tumor-cell phenotype behavior (e.g., proliferation, differentiation, and adhesion), but also contribute to the control of the local microenvironment, immune responses, and metastasis. Therefore, these glycans serve as ligands for cells in the tumor microenvironment through endogenous lectins.

## Siglecs

Sialic acid-binding immunoglobulin superfamily lectins (siglecs) are the largest family of sialic-acid-binding molecules (93–95). Siglecs are expressed on specific subpopulations of hematopoietic cells where they exert their immune-regulatory function. Many siglecs contain intracellular tyrosine motifs, which include one or more membrane-proximal immunoreceptor tyrosine-based inhibitory motif (ITIM) and a membrane-distal ITIM-like motif (93, 94). These motifs are involved in inhibitory signal transduction. Based on both sequence similarity and conservation between mammalian species siglecs are divided in two major subgroups. The first group comprises Siglec-1 (sialoadhesin, CD169), Siglec-2 (CD22), Siglec-4 (myeloid-associated glycoprotein), and Siglec-15. The second subfamily of CD33/Siglec-3 related siglecs consists of 10 human members (Siglec-3, -5, -6, -7, -8, -9, -10, -11, -14, and -16) and 5 rodent members (Siglec-3, -E, -F, -G, and -H) (93, 95). The first subgroup with its evolutionary conserved members has restricted expression patterns. For instance Siglec-1 is specifically expressed on macrophages, Siglec-2 on B-cells and Siglec-4 on oligodendrocytes and Schwann cells in the nervous system (96). On the other hand, CD33-related siglecs display a more divergent expression pattern dependent on developmental stage of immune cells (93, 95). The high sialic acid concentration on the cell-surface of siglec-expressing cells often leads to binding to the cell glycans (in cis) or adjacent cells (in trans). Siglecs can be affected by various stimuli including cytokines, toll-like receptor activation, and viral and bacterial infections, the biology of siglecs is therefore rather complex (96). The binding specificity of siglecs depends on the distinct types, linkages (α2,3, α2,6, and α2,8), arrangements of sialic acids, their way of presentation on different cells, organs, and organisms. Siglec binding to ligands modulates cell–cell interactions, cell proliferation, cell death, and endocytosis (96–99).

## The Role of Siglecs in Cancer Progression

Accumulating evidence indicates that the interaction between tumor-specific glycans and lectins on immune cells are involved in modulation of the tumor microenvironment (100). The inhibitory nature of siglec upon binding of specific glycan may lead to dampening of immune responses and thereby escape of immune surveillance and clearance. Whether siglecs contribute to cancer progression through recognition of distinct cancer-specific glycan structures is currently under investigation. Non-malignant colon epithelial cells express di-sLe^a^ epitopes that serve as ligands for both Siglec-7 and -9 (15). The expression of siglec ligands was decreased upon malignant transformation, which was associated with enhanced expression of sLe^x^ and sLe^a^ epitopes (26). Expression of ST6GalNAc6, which synthesizes di-sLe^a^ in human colon cancer cells resulted in increased di-sLe^a^, loss of sLe^a^ epitopes, and increased binding to Siglec-7 (41). Mainly resident macrophages were found to carry Siglec-7 and -9 in a colonic lamina propria and Siglec-7/9 ligation could suppress macrophage-mediated cyclooxygenase-2 (COX2) and prostaglandin E2 expression and thereby prevent inflammatory damage of the colonic mucosa (15). Siglec-15, which preferentially recognizes sTn antigen, is expressed in tumor-associated macrophages (TAMs) in various human carcinoma tissues including lung, liver, and rectum (101). Binding of myeloid cells through Siglec-15 to sTn on tumor cells resulted in increased TGF-β secretion into the tumor microenvironment that is associated with cancer progression. Interestingly, Siglec-15 expression was induced by M-CSF, which usually polarizes macrophages to M2 phenotype commonly detected in the tumor microenvironment.

Siglec-1 is expressed in a subset of macrophages that are involved in the pathophysiology of cancer (102). Clinical observation showed that increased Siglec-1 is present in splenic marginal cell lymphoma as well as in macrophage infiltrates of MUC1-positive breast cancers (103, 104). Siglec-1 positive macrophages were found to infiltrate into rat xenograft tumors in a CCL2-dependent manner (105). On contrary, recent study demonstrated that Siglec-1 positive macrophages in regional lymph nodes of colorectal carcinoma patients promote CD8^+^ T-cell mediated anti-tumor immunity and are associated with a better prognosis for these patients (106).

Siglec-9, a surface receptor on NK cells, B-cells, and monocytes, has been identified as a receptor for mucin MUC16 (14). Cell-surface bound as well as soluble MUC16 is overexpressed in human ovarian tumor cells and detected in peritoneal fluid of cancer patients (107). Engagement of Siglec-9 on monocytes also induced secretion of immunosuppressive cytokine IL-10 (108). Similar immune-suppression mediated by Siglec-7 on NK cells was observed in renal cell carcinoma expressing disialosyl globopentaosylceramide (DSGb5) as a major ganglioside (109). Recent study from C. Bertozzi group provided strong evidence that siglec-7-mediated cytotoxicity of NK cells can be modulated by the alteration of glycans on cell surfaces (110). Presentation of sialylated ligands on tumor cells recognized by siglec-7 resulted in enhanced phosphorylation of cytoplasmic tyrosine residues, causing dampening of cytolytic activity.

The association between Siglec-9 positive immune cells and MUC1-positive tumor cells has been detected in tissues of human colon, pancreas, and breast cancer. Interestingly, Siglec-9 binding to MUC1 expressing tumor cells was shown to induce recruitment of β-catenin in tumor cells resulting in promotion of cell growth *in vitro* (111). These findings suggest that Siglec-9 engagement of carcinoma mucin MUC1 may be involved in tumor growth, however; the nature of Siglec-9 ligands as well as the cellular context *in vivo* remains to be defined.

Taken together, the current evidence is largely based on clinical correlation of cancer–glycan expression and several experiments showing Siglec-cancer–glycan interaction *in vitro*. Whether these interactions indeed functionally modulate immune cell responses in the tumor microenvironment and thereby affect cancer progression *in vivo* requires experimental validation.

## Siglecs as Target of Cancer Therapy

The identification of Siglec-2 and Siglec-3 as markers of acute myeloid leukemia (AML) and B-cell lymphomas raised interest in potential immunotherapy (112–114). Anti-Siglec-2 and siglec-3 specific antibodies were conjugated with variety of toxins and such immunotoxins have been targeted in several autoimmune diseases and hematological malignancies [reviewed in Ref. (93, 94, 115)]. In the majority of acute lymphoblastic leukemias (ALL) Siglec-2 (CD22) was identified as a useful target for cell-depletion therapy (116). Inotuzumab ozogamicin is an immunotoxin comprised of a humanized IgG4 monoclonal antibody covalently linked to calecheamicin (CMC-544). CMC-544 was active against B-cell tumors in preclinical models and has been evaluated in phase I study for patients with B-cell lineage ALL (117). Inotuzumab ozogamicin used as a single therapy in patients with refractory-relapsed ALL showed positive results.

The immunotoxin gemetuzumab ozogamicin (OG, Mylotarg; Wyeth, Madison, NJ, USA), which consists of a humanized anti-CD33 (siglec-3) murine antibody linked to calicheamicin, was approved by the FDA for treatment of CD33+ AML patients. Binding and endocytosis of the conjugate resulted in the intracellular release of the toxin causing cell death of CD33+ cells (94, 115). However the drug is off the market since 2010 because the key phase III trial (South West Oncology Group Study S0106) in which GO was combined with induction chemotherapy failed to improve disease-free survival and caused higher fatal induction toxicity rate compared to chemotherapy alone (118). Recent studies using lower or fractionated dose of GO suggest that GO may still improve survival of distinct subsets of AML patients, particularly patients with favorable cytogenetics (119). New approaches with humanized CD33 antibody conjugated to synthetic DNA cross-linking pyrrolobenzodiazepine (SGN-CD33A) have been developed and revealed promising effectiveness in animal models (120). SGN-CD33A is now currently being tested in a phase I trial (ClinicalTrials.gov: NCT01902329).

## Galectins

In contrast to siglecs and selectins, which are mostly cell-surface-bound receptors, galectins are soluble immunomodulatory lectins (121). Galectins bind to galactose that is either β1,3- or β1,4-linked to *N*-acetylglucosamine, a common disaccharide found both on *N*- and *O*-linked glycans and glycolipids. Galectins act both intracellularly by modulating signaling pathways and extracellularly as regulatory receptors (100). Up to date the galectin family consists of 15 members, which are classified into three groups based on structural differences: prototype galectins (Galectin-1, -2, -5, -7, -10, -11, -13, -14, and -15) having one carbohydrate recognition domain (CRD), tandem repeat-type galectins (Galectin-4, -6, -8, -9, and -12) having two CRDs, and the single member Galectin-3, which has one CRD connected to a non-lectin N-terminal region responsible for oligomerization (100). Galectins are expressed by various cell types including epithelial and immune cells, but their expression is altered during progression of colon, breast, lung, pancreatic, head and neck, and cervical cancers (16, 122). Many studies indicate that cancer-associated galectins could regulate cancer cell proliferation, signaling, adhesion, invasion, and metastasis (122–124). Galectin-1 and Galectin-3 were most intensively studied in context of cancer.

## Galectin-1

Accumulating evidence indicate that tumor-derived Galectin-1 contributes to immunosuppressive activity in different tumors, including lung and pancreatic carcinoma, melanoma, and neuroblastoma (16, 125–127). It has been shown that Galectin-1 binding to T-cells through *N*- and *O*-linked glycans on CD43 or CD45 mucins induces apoptosis of activated T-cells (128, 129). Galectin-1 expression by melanoma cells induced apoptosis of tumor-specific effector T-cells, and Galectin-1 inhibition allowed generation of a tumor-specific T1 response (126). Modification of cell-surface glycosylation affects glycan pattern on T-cells and thereby changes Galectin-1 binding. Enhanced expression of α2,6-sialyltransferase-1 (ST6Gal1) selectively modified *N*-glycans on CD45 and thereby inhibited Galectin-1 binding (130). How Galectin-1 contributes to immune-suppression in tumors has been delineated in lung cancer (131). High expression of Galectin-1 in lung cancer cell lines, as well as in human tumor tissues, alters the phenotype of monocyte-derived dendritic cells and impairs T-cell response, concomitant with increased presence of regulatory T-cells (Tregs). The regulatory effect of Galectin-1 is mediated by increased expression of IL-10 in monocytes thereby inducing a Th2-dominant cytokine profile. The enhanced infiltration of CD11c^+^ dendritic cells in human lung cancer samples has been recapitulated in a mouse model, which was completely omitted after transplantation of Galectin-1 silenced tumor cells. In another study, the amount of Galectin-1 positive cells correlated with the tumor grade in human breast cancer (132). Silencing of Galectin-1 in a metastatic murine mammary tumor led to a reduction of tumor growth and lung metastasis with a concomitant reduction in infiltrating regulatory T-cells.

Experimental evidence also suggests that Galectin-1 expressed on various tumor-cell types including hepatocellular carcinoma, melanoma, ovarian, and prostate cancer cells mediates tumor-cell adhesion to the extracellular matrix (133, 134). In addition, Galectin-1 mediated attachment of cancer cells to the extracellular matrix and endothelial cells through binding to CD44 and CD326 on murine breast and colon cancer cells (16). Galectin-1 might also be involved in formation of platelet-cancer cell complexes since it was shown to activate platelets (135). Murine breast, colon, and Lewis lung cancer cells with silenced Galectin-1 showed decreased lung metastasis, which was associated with increased T-cell numbers and reduced angiogenesis (16, 125). Taken together, tumor-derived Galectin-1 exerts its immunosuppressive function through binding to endogenous (non-tumor-derived) glycans and thereby contributes to cancer progression.

## Galectin-3

There is accumulating evidence that the cancer-associated T, Tn, and sTn structures promote metastasis through binding to Galectin-3. Galectin-3 expression is also increased in patient sera of several cancer types and associated with increased risk of metastasis (136, 137). For instance, T antigen expression by breast and prostate cancer cells facilitated interactions with cancer-associated Galectin-3 or with endothelial associated Galectin-3 (66, 138–140). These interactions lead to homotypic aggregation of cancer cells, which protects cancer cells from apoptosis induced by the lack of adhesion to the extracellular matrix (139). In addition, cancer cell-associated T antigens can induce Galectin-3 expression on the endothelium, which enabled cancer-endothelium adhesion (140). Another study has shown that lysosomal-associated membrane protein-1 (LAMP-1) on highly metastatic melanoma cells carries *N*-acetyllactosaminyl structures, which are recognized by Galectin-3 on lung endothelial cells suggesting that lung endothelial galectin-3 can serve as anchor for LAMP-1 expressing tumor cells in the circulation (141).

A characteristic feature of galectins is the induction of complex formation by cross-linking glycoproteins, which can form multimers “lattice” microdomain (121). Complex *N*-glycans are formed by GnT5 modification of *N*-glycans that are the ligands for Galectin-3 (142). Expression of GnT5 has long been implicated in tumor progression and metastasis (17). In particular, the absence of GnT5 delayed tumor formation and suppressed metastasis (21). Accordingly, up-regulated GnT5 expression has been observed in various human cancers (18, 143); and the ectopic expression of the GnT-V in multiple epithelial cells resulted in increased cell motility, tumor formation, and enhanced metastasis (144, 145). Furthermore, GnT5-dependent modifications of tyrosine kinase receptors such as EGF, TGF-β, IGFR, and PDGF enhanced affinity to galectin-3 and thereby prolonged their cell-surface expression (22, 146). Galectin-3-induced lattice formation prevented the surface clearance of receptors by clathrin-dependent endocytosis and enabled interaction with inhibitory caveolin-1 domains.

Branched *O*-glycans with poly-*N*-acetyllactosamine structures are recognized by Galectin-3 (147). In C2GnT1-expressing bladder tumor cells core 2 *O*-glycans present on MHC class I-related chain A are bound to Galectin-3 that reduced the affinity for the activating NK cell receptors NKG2D, thereby impairing NK cell function and anti-tumor activity.

Recent findings suggest that Galectin-3 also regulates dynamics of N-cadherin and the lipid raft marker ganglioside GM1 (148). Accumulation of N-cadherin and GM1 at cell–cell junctions destabilized cell–cell junctions and thereby promoted tumor-cell migration. *N*-glycans on α5β1 integrin are important for their proper binding to fibronectin (149, 150). Increased GnT5 mediated β1,6-branching reduces cell-surface clustering of α5β1 integrin, specifically of the β1 subunit, resulting in a less adhesive phenotype due to reduced adhesion to fibronectin and modulates fibronectin matrix remodeling in tumors (20, 151). Thus, Galectin-3 lattice formation provides another mechanism how altered glycosylation contributes to the malignant and invasive phenotype of tumor cells (148).

## Selectins

Selectins are vascular cell adhesion molecules that belong to a family of C-type lectins, which facilitate the initial attachment of leukocytes to the endothelium during the process of leukocyte extravasation. The selectin family consists of L-, E-, and P-selectin, which share around 50% sequence homology in their C-type lectin domain (152). L-selectin (LECAM-1 and CD62L) is constitutively expressed on almost all hematopoietic cell types including myeloid cells, naïve, and some activated memory T-cells (152) and enables adhesion of leukocytes to the activated endothelium or in high endothelial venules of the peripheral lymph nodes (153, 154). E-selectin (ELAM-1 and CD62E) is exclusively displayed on endothelial cells, which requires *de novo* expression in response to inflammatory stimuli such as TNF-α and Il-1β. However, skin and parts of the bone marrow microvasculature have been shown to constitutively express certain E-selectin levels (155). On contrary, P-selectin (PADGEM and CD62P) is stored in alpha-granules of platelets as well as in Weibel–Pallade bodies of endothelial cells and can be rapidly mobilized to the cell-surface upon activation of platelets or the endothelia. E- and P-selectin bind to ligands on myeloid cells (156), certain types of lymphocytes (152) but also to several types of tumor cells (157–159). Selectins are the most-studied lectins in cancer biology, which promote cell–cell interaction with tumor cells and their microenvironment (9). All three selectins have been shown to contribute to tumor dissemination and specifically facilitate processes when the tumor cells are in the circulation.

## Selectin Ligand Expression Correlates with Cancer Progression

There is compelling clinical and experimental evidence that overexpression of tetrasaccharides sLe^x^ and sLe^a^ correlates with poor prognosis due to enhanced metastatic phenotype in a number of cancer types, including colon, gastric, prostate, renal, pancreatic, and lung cancer (89, 160–165). Enhanced expression of sLe^x/a^ on cancer cells correlated with increased ability to adhere to E-selectin or to the activated endothelial cells and stromal cells *in vitro* (157, 166–168). Furthermore, high cell-surface expression levels of sLe^x^ were linked to enhanced metastatic activity in various experimental metastasis models using human carcinoma cells compared to lower or minimal sLe^x^ expression (169–171).

**Figure 2:**
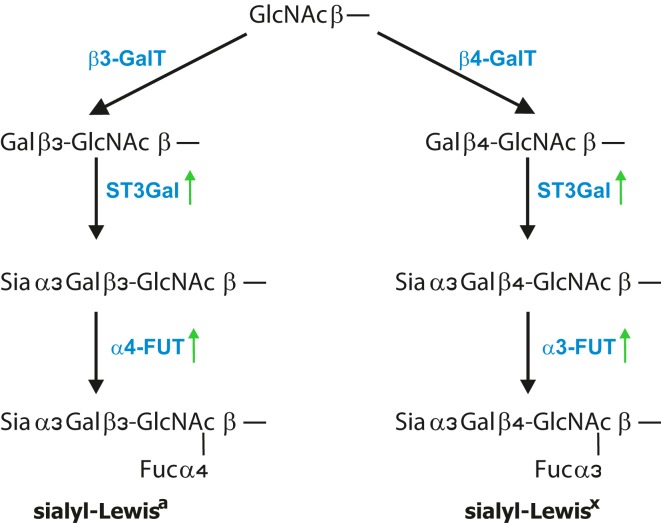
**Formation of Lewis antigens**. Terminal GlcNAc residues, particularly on core 2 structures, are further extended by addition of β1,4 galactose, for Lewis^x^ epitope, and β1,3 galactose, for Lewis^a^ epitope. This is further followed by the addition of α2,3-linked sialic acid to Gal by ST3Gal enzymes and finalized by the addition of α1,3-linked fucose for sLe^x^ and α1,4-linked fucose for the sLe^a^ antigen. FUT3 finalized the synthesis of Lea antigen, while FUT6 and FUT7 were shown to finalize Le^x^ epitopes.

The minimal recognition motif for all three selectins are tetrasaccharides sLe^x/a^ (Figure 2) (172). SLe^x^ are terminal structures of *N*-or *O*-linked glycans attached to glycoproteins and glycolipids displayed by most circulating leukocytes and endothelial cells whereas sLe^a^ is detected on some epithelial cells but mostly on various tumor cells (3, 4, 173). The four glycosyltransferases *N*-acetylglucosaminyltransferase, β1,4-galactosyltransferase, α2,3-sialyltransferase, and α1,3-fucosyltransferase-7 are responsible for synthesis of sialyl-Lewis^a/x^ structures on cells of the hematopoietic system (172, 174). Efficient selectin binding to carbohydrates usually requires a glycoprotein scaffold that facilitates the presentation of selectin ligands in clusters (175). One of the best characterized ligands for all three selectins is the P-selectin glycoprotein ligand-1 (PSGL-1), which is concentrated on the tips of microvilli on leukocyte surface (176). To the most common mucins carrying selectin ligands that are associated with cancer progression belong MUC1, MUC2, MUC4, and MUC16 (35, 45, 177, 178). Apart from mucins, several other selectin ligand carriers on tumor cells have been identified that includes CD24, CD44, death-receptor 3, E-selectin ligand-1, PSGL-1, and podocalyxin-like protein and this list is by far not complete (179–183). Several of these ligands are also expressed on tumor cells and are associated with cancer progression. For instance, CD44 glycoproteins exist in several isoforms and are expressed on epithelial and endothelial cells as well as on multiple cancer cell types such as gastric, colorectal, pancreatic, and lung cancer (184–186). The aberrant expression of CD44 in colorectal carcinoma cells correlated with increased metastatic potential *in vivo* (187, 188). Based on flow-based adhesion assays *in vitro*, CD44v on human colon carcinoma cells binds to P-, E-, and L-selectin (189, 190). The majority of selectin ligands are presented on mucins, but they can be found equally functional also on *N*-linked glycans or glycolipids. Finally, P- and L-selectins also bind to heparin, heparan sulfate, and sulfated glycolipids, which also indicates certain flexibility in ligand recognition (9, 175). In addition, chondroitin sulfate glycosaminoglycans (CS-GAGs) on breast cancer cells were identified to serve as a P-selectin ligand that is associated with breast cancer metastasis (191). Despite the large variety of glycans, tumor cells express sialylated and fucosylated molecules, mostly on mucins which are also recognized by selectins (158, 159, 167, 192, 193).

Increased expression of sLe^x/a^ in tumor cells has been attributed to elevated levels of α1,3-fucosyltransferase-7 (FUT7), which has also been shown to correspond with increased malignancy in lung cancer patients (161). In addition, overexpression of α1,3-fucosyltransferase-3 and -6 in metastatic prostate cancer cells correlated with higher sLe^x^ levels and more metastasis that was dependent on E-selectin-mediated recruitment to distant sites (169, 194). Genes encoding for FUT3, FUT4, and ST3GAL6 enzymes that are involved in sLe^x^ synthesis were significantly increased in breast cancers and correlated with metastasis to the bone where sLe^x^ receptor E-selectin is constitutively expressed (195). Inflammatory cytokines might also be involved in sLe^x^ production. TNF-α enhanced motility and invasion properties of prostatic cancer cells were associated with selective upregulation of genes related to sLe^x^ synthesis (196). Studies analyzing prostate and pancreatic cancer cell homing into bone showed that E-selectin-mediated adhesion is dependent on enhanced α1,3-fucosyltransferase, FUT3, FUT6, and FUT7 activity (197, 198). Consequently, down-regulation of α1,3-fucosyltransferase activity dramatically reduced prostate cancer incidence. However, there is also the possibility that selectin-mediated activation of either tumor cells or the tumor microenvironment further promote inflammation that is a hallmark of cancer progression.

## P-Selectin

The association between circulating cancer cells, platelets, and formation of tumor microemboli is widely accepted (199–202). Many studies showed that platelets enhance hematogenous dissemination, intravascular tumor-cell survival, and metastasis (203–206). However, the major mechanism of platelet-adhesion to tumor cells has been found to be mediated by platelet P-selectin (6). Platelet-tumor cell interactions were significantly reduced in P-selectin deficient mice, and consequently attenuation of metastasis was observed. Enzymatic removal of carcinoma mucins carrying selectin ligands from tumor cells prior to tail vein injection resulted in attenuated metastasis comparable to the absence of P-selectin (158, 203). In addition, endothelial P-selectin-mediated interactions also contributed to metastasis indicating that both platelet and endothelial P-selectin promote early events during tissues colonization (11, 207). Another study shows that platelets promote lung metastasis of B16F1 melanoma and 4T1.2 breast cancer cells (208). Platelet depletion resulted in a significant reduction of lung metastasis when compared to NK cell depleted animals, indicating an additional pro-metastatic function of platelets. These findings are in agreement with a direct effect of platelet–tumor-cell interactions that promotes the metastatic behavior of tumor cells (209). Taken together, P-selectin-mediated interactions significantly contribute to the early steps of metastasis when tumor cells are in circulation.

## L-Selectin

L-selectin binds to a variety of tumor cells and contributes to metastasis (167, 210). Intravenous injection of human and murine tumor cells in L-selectin deficient mice resulted in reduced recruitment of leukocytes and subsequently attenuated metastasis that confirmed the active role of L-selectin-mediated interaction in this process (11, 13). Metastasis was further attenuated in P- and L-selectin double deficient mice providing evidence that both selectins synergistically contribute to metastasis (11). In addition, the enhanced expression of selectin ligands around the metastatic tumor cells was detected with L-selectin chimera, which correlated with the recruitment of leukocytes (13). These findings indicated that L-selectin is either responsible for recruitment of leukocytes or their interactions within the metastatic microenvironment. Enhanced presence of inflammatory cells, primarily myeloid-derived cells, in the tumor microenvironment is usually associated with tumor growth and metastatic dissemination (211, 212). Thus, L-selectin represents a potential facilitator of myeloid cell recruitment to metastatic sites and thereby promotes early steps of metastasis, e.g., tumor-cell extravasation (13, 213). During inflammation, leukocyte interaction with the endothelium results in induced vascular permeability. However, whether L-selectin promotes metastasis through a direct engagement with selectin ligands on tumor cells or rather mimics inflammatory-like reaction accompanying the process of tumor-cell seeding in distant organs remains to be determined.

## E-Selectin

E-selectin has been the first selectin intensively studied in context of metastasis (9, 10). The original hypothesis was that E-selectin mediates metastatic dissemination to distant organs through binding to ligands on tumor cells, similarly to leukocyte adhesion during inflammation (3). Numerous studies provided evidence that tumor cells expressing selectin ligands adhere to activated endothelium under flow condition *in vitro* (157, 168, 181). While different E-selectin ligands were linked to enhanced metastasis, the majority of them belong to the mucin type molecules. Despite the observation of increased primary tumor growth in selectin deficient mice, which seems to be linked to reduced anti-tumorigenic infiltration of immune cells (214), there is accumulating evidence that E-selectin promotes cancer metastasis in animal models. Enhanced E-selectin expression was observed in the liver during metastatic colonization and the down-regulation of E-selectin resulted in attenuation of metastasis (215, 216). Metastasis was redirected to the E-selectin overexpressing liver using experimental lung metastasis model, which provided direct evidence for involvement of E-selectin in facilitation of tumor-cell seeding (217). Accordingly, experimental liver metastasis of human colon carcinoma cells was also E-selectin-dependent (218). However, experimental lung metastasis of human colon adenocarcinoma cells remained unchanged in E-selectin deficient mice (219). On contrary, spontaneous metastasis of human breast cancer cells to the lungs was significantly attenuated in E-selectin-deficient mice (220). Interestingly, Hiratsuka et al. showed that factors secreted from primary tumors can activate endothelial focal adhesion kinase and E-selectin expression in the lung vasculature and thereby induce the formation of permissible sites for metastasis (221). Enhanced homing of metastatic tumor cells to these sites was observed and was associated with metastasis. These observations indicate that primary tumors can actively form a distant metastatic niche and upregulate expression of cell adhesion molecules involved in tumor cell-endothelial interactions. In conclusion, there is convincing evidence that endothelial E-selectin facilitates metastasis by enabling tumor-cell adhesion to vasculature. Nevertheless, the exact mechanism of E-selectin facilitation of metastasis remains to be defined.

## Carcinoma Mucins as Initiators of Cancer-Related Prothrombotic Activity

Altered cancer glycosylation is not reflected only on cell-surface molecules, but aberrantly glycosylated proteins are detected in the circulation (26). Antibodies raised against tumor cells, were shown to specifically recognize glycan structures, e.g., sLe^a^, which are currently used for cancer diagnostics (45). The presence of carcinoma mucins (e.g., CA-125, CA19-9), which are shedded from tumors, are routinely used as serum tumor markers in diagnosis of cancer. Besides, efficient binding of recombinant soluble selectin to carcinoma mucins has been observed (158, 222). Increased thromboembolism is a recognized complication in various carcinomas, particularly mucinous carcinomas, however; there are several pathologic mechanisms likely to be involved (7). Idiopathic thromboembolism, which is frequently associated with occult carcinomas, belongs to the Trousseau syndrome. Recent studies provided evidence that intravenous injection of carcinoma mucins carrying selectin ligands into mice resulted in generation of platelet-rich microthrombi (222). This pathology was markedly diminished in P-selectin or L-selectin deficient mice. Interestingly, carcinoma mucins could not activate platelets and thereby could not generate microthrombi in mice lacking PSGL-1 (223). Carcinoma mucins initiated thrombosis only in the presence of platelets that induced release of cathepsin G from neutrophils through a selectin-dependent, reciprocal activation of neutrophils and platelets. Taken together, carcinoma mucins carrying selectin ligands in blood circulation may serve as initiators of thrombi formation observed in cancer patients.

## Selectins Shape the Metastatic Microenvironment

There is accumulating evidence that selectins facilitate heterotypic interactions between tumor cells and blood components, including the endothelium and thereby promote tumor-cell seeding, survival and extravasation (8, 9, 224). When circulating tumor cells arrest in the microvasculature of distant organs, early on markers of endothelial cell activation and inflammation, including E-selectin, were upregulated in experimental lung and liver metastasis models (219, 225–228). Enhanced E-selectin expression was detected also in the metastatic lungs using a spontaneous metastatic model with Lewis lung carcinoma (219, 221). Consequently, inhibition of endothelial activation and/or E-selectin function attenuated metastasis (227, 229). Endothelial activation caused by factors derived from primary tumor or from arrested tumors in the vasculature promoted selectin-mediated interactions and formation of a permissive microenvironment within the vasculature prior to tumor-cell extravasation (11, 13, 213). Tumor-cell glycan-induced and P-selectin-dependent endothelial activation resulted in enhanced expression of E-selectin and vascular cell adhesion molecule 1 (VCAM-1) and promoted lung colonization and metastasis (213). In addition, elevated production of chemokine CCL5 contributed to the recruitment of monocytes. Accordingly, endothelial VCAM-1 expression was induced by tumor-cell embolus that resulted in increased recruitment of myeloid cells supporting metastasis (225). Recruitment of inflammatory cells, especially myeloid-derived cells, is strongly associated with enhanced metastatic colonization that is at least partially dependent on L-selectin (12, 13, 213, 230–232). Taken together, the selectin-mediated interactions play a critical role during the establishment of metastasis that is co-initiated by aberrant glycans on tumor cells in circulation. Whether tumor glycans only initiate the inflammatory-like cascade leading to metastasis or have further function in shaping this process remains to be defined.

## Conclusion and Perspectives

Cancer-associated aberrant glycosylation has been identified in virtually every type of cancer. Expression of cancer-specific glycan epitopes represents a great opportunity to explore them for diagnostics and potentially specific targeting of tumors. Considering that genes only indirectly regulate glycan formation, it is still puzzling that glycan epitopes have been consistently validated as cancer markers. Based on the broad expression and high specificity for cancer tissues, T antigen is currently explored as a potential target for the development of cancer diagnostics and immunotherapeutics (16, 233). Since the expression of sTn antigens on the majority of tumors correlated with poor prognosis, the sTn antigen has become a target for cancer vaccine (58, 61). Administration of sTn disaccharide conjugate to highly immunogenic protein induced antibodies against sTn and showed protective effects in a mouse model of breast cancer (234). Although a randomized phase III clinical trial using the same sTn vaccine did not improve overall survival, patients with high titer against the sTn had significantly prolonged overall survival (235).

The accumulating knowledge about the function of lectin–tumor-cell glycan interactions in cancer will open ways for new approaches to interfere with cancer progression. However, the exploitation of such therapeutic opportunities requires a comprehensive knowledge about the underlying mechanisms of lectin-mediated interactions. Nevertheless, the role of selectins in cancer progression has been extensively investigated in number of preclinical models and the mechanism at least partially characterized (9). Clearly, further studies in the exact mechanism of action are still required, but selectin inhibition in cancer has been inadvertently clinically tested in cancer patients treated with antithrombotic therapies (236). Unfractionated heparin as well as low molecular weight heparin has a strong P- and L-selectin inhibitory activity at clinically relevant concentrations. Retrospective analysis of clinical studies revealed that apart from antithrombotic activity, heparin improved survival of cancer patients especially in patients with early stage disease. Still, prospective and well-designed clinical study remains to be performed. Similarly, development of highly specific ligand probes for siglecs (e.g., Siglec-2) revealed the ability to target siglec-expressing cells (94). Further investigations are required for deciding whether glycan-specific targeting of lectins involved in cancer modulation (e.g., siglec, selectins, or galectins) or rather development of glycan-specific targeting of tumor cells represents the right approach for the treatment of cancer. The cell-surface presentation of unique glycan epitopes makes them an “ideal” candidate for targeting since they are both specific and therapeutically accessible. Future studies need to validate the therapeutic potential in clinically relevant experimental models prior to clinical evaluation.

## Conflict of Interest Statement

The authors declare that the research was conducted in the absence of any commercial or financial relationships that could be construed as a potential conflict of interest.
